# Efficacy of Immunotherapy in Patients with Metastatic Mucosal or Uveal Melanoma

**DOI:** 10.1155/2018/1908065

**Published:** 2018-12-02

**Authors:** Claire Mignard, Aurélie Deschamps Huvier, André Gillibert, Anne Bénédicte Duval Modeste, Caroline Dutriaux, Amir Khammari, Marie-Françoise Avril, Nora Kramkimel, Laurent Mortier, Pierre Marcant, Thierry Lesimple, Caroline Gaudy-Marqueste, Candice Lesage, Laurent Machet, François Aubin, Nicolas Meyer, Nathalie Beneton, Géraldine Jeudy, Henri Montaudié, Jean-Philippe Arnault, Laetitia Visseaux, Sabiha Trabelsi, Mona Amini-Adle, Eve Maubec, Yannick Le Corre, Dan Lipsker, Ewa Wierzbicka-Hainaut, Noémie Litrowski, Andreea Stefan, Florence Brunet-Possenti, Marie-Thérèse Leccia, Pascal Joly

**Affiliations:** ^1^Dermatology Department, Institute for Research and Innovation in Biomedicine, INSERM 1234, Rouen University Hospital, University of Normandie, Rouen, France; ^2^Department of Biostatistics, Rouen University Hospital, Rouen, France; ^3^Department of Dermatology, Oncology Unit, Saint André Hospital, Bordeaux University Hospital, France; ^4^Onco-Dermatology Department, CHU Nantes, CRCINA, CIC1413, University Nantes, Nantes, France; ^5^APHP, Department of Dermatology, Cochin Hospital and University Paris Descartes, 89 rue Assas, 75006 Paris, France; ^6^Department of Dermatology, Hôpital Huriez, Lille University Hospital, 59045 Lille, France; ^7^Department of Medical Oncology, Eugene Marquis Center, Rennes, France; ^8^Department of Dermatology and Skin Cancer, La Timone Hospital, 265 rue St Pierre, 13885 Marseille Cedex 05, France; ^9^Department of Dermatology, Montpellier University Hospital, 34295 Montpellier, France; ^10^Department of Dermatology, CHRU de Tours et Université François Rabelais de Tours, France; ^11^Department of Dermatology, EA3181, Besancon University Hospital, France; ^12^Department of Dermatology, Toulouse III University – Paul Sabatier, Institut Universitaire du Cancer and Toulouse University Hospital, France; ^13^Department of Dermatology, Le Mans hospital, France; ^14^Department of Dermatology, Dijon University Hospital, France; ^15^Department of Dermatology, Nice University Hospital, INSERM, U1065, Centre Méditerranéen de Médecine Moléculaire, Team 12, Nice, France; ^16^Department of Dermatology, Amiens-Picardie University Hospital, 80054 Amiens Cedex 1, France; ^17^Department of Dermatology, Reims University Hospital, France; ^18^Department of Dermatology, Grenoble University Hospital, France; ^19^Department of Dermatology, Hospices Civils de Lyon, Lyon 1 University, Centre Hospitalier Lyon Sud, 69495 Pierre Bénite Cedex, France; ^20^APHP, Avicenne Hospital, Department of Dermatology, University Paris 13, Bobigny, France; ^21^Department of Dermatology, Angers University Hospital, UNAM, France; ^22^Faculty of Medicine, University of Strasbourg and Department of Dermatology, Strasbourg University Hospital, France; ^23^Department of Dermatology, Poitiers University Hospital, France; ^24^Department of Dermatology, Monod General Hospital, le Havre, France; ^25^Department of Dermatology, Caen University Hospital, France; ^26^Department of Dermatology, Bichat University Hospital, Paris, France

## Abstract

**Background:**

The objective was to assess the response rate and survival of patients with metastatic mucosal melanoma (MM) and uveal melanoma (UM) treated with anti-CTLA-4 or anti-PD-1 monoclonal antibodies (mAbs).

**Methods:**

A multicenter retrospective study was performed in 25 dermatology departments in France. All patients with stage III-C to IV MM or UM who were treated with anti-CTLA-4 or anti-PD-1 mAbs between 2008 and 2016 were included and compared after adjustment for main prognostic factors with a second cohort of patients treated with chemotherapy. Tumor response was evaluated according to RECIST v. 1.1 criteria at Week 12.

**Results:**

Four-hundred-and-thirty-nine patients were included, 229 MM (151 immunotherapy, 78 chemotherapy) and 210 UM (100 immunotherapy, 110 chemotherapy). Response rates of MM patients treated with immunotherapy were 18/151 (11.9%; 95% CI:7.2%-18.2%), versus 11/78 (14.1%, 95% CI:7.3%-23.8%) in patients treated with chemotherapy (p=0.87). No tumor response was observed in UM patients treated with immunotherapy, versus 4/110 responses (3.6%, 95% CI:1.0-9.0%) in patients treated with chemotherapy (p=0.15). The adjusted overall survival (OS) of MM patients treated with immunotherapy was longer than that of patients treated with chemotherapy HR=0.62 (95% CI: 0.43-0.91), p=0.014, with an unadjusted median OS of 15.97 months [interquartile range (IQR)=6.89-27.11] and 8.82 months [IQR=5.02-14.92], respectively. The adjusted OS of UM patients treated with immunotherapy was not significantly different from that of patients treated with chemotherapy (HR=0.98, 95% CI: 0.66–1.44) p=0.92, with an unadjusted median OS of 13.38 months [IQR=6.03-29.57] and 11.02 months [IQR=6.13-23.93], respectively.

**Conclusion:**

Immunotherapy significantly improves OS for MM. The prognosis of metastatic UM remains poor.

## 1. Introduction

Mucosal melanoma (MM) and uveal melanoma (UM) are rare types of melanoma, corresponding to between 4 and 6.8% of melanoma in Caucasians [1–4]. Mucosal melanomas include melanomas located in the sinonasal and oral cavity (50%), anorectal region (25%), urogenital tract (20%), and conjunctiva [5, 6]. Uveal melanomas include melanomas occurring on the choroid, ciliary body, and iris. The prognosis of MM and UM is considered poorer than that of skin melanomas, since they are often diagnosed at an advanced metastatic stage and have particular clinical and genetic characteristics [7].

Despite these features, treatment options proposed in patients with metastatic MM and UM are the same as those in patients with skin melanoma. Chemotherapy is poorly effective in MM and even less in UM with response rates ranging from 0 to 15 % [3, 8–10]. Three immune checkpoint inhibitors have been approved in the treatment of patients with metastatic cutaneous melanoma: ipilimumab, an anti-CTLA-4 monoclonal antibody (mAb), and nivolumab and pembrolizumab, which are both directed against the programmed cell-death protein 1 (PD1). Recent studies showed that these drugs improve the prognosis of patients with metastatic cutaneous melanoma. Response rates of 10.9% to 15.2% have been reported with anti-CTLA-4 and of 19% to 52% with anti-PD-1 mAbs [11, 12]. Additionally, these latter molecules improved patients' survival, with one- and two-year overall survival rates of 68.4% to 72.9% with anti-PD-1 mAbs and of 43% to 55% with anti-CTLA-4 [13–15].

Since UM and MM are quite uncommon, the efficacy of anti-CTLA-4 and anti-PD-1 mAbs has not been specifically evaluated in large series of patients with metastatic MM and UM [16].

Rather low response rates between 5 and 17% have been reported with anti-CTLA-4 in MM, corresponding to median overall survival durations between 6.4 and 9.6 months [17–20]. To the best of our knowledge, only a few studies have assessed the efficacy of anti-PD1 mAbs in limited series of patients with MM or UM [21, 22]. The aim of the present study was to assess the response rate, and overall and progression-free survival in a large multicenter retrospective series of patients with metastatic MM and UM treated with anti-CTLA-4 or anti-PD-1 mAbs. To compare these results with those previously obtained using chemotherapy regimens, we also assessed the response rate and survival in a series of patients who were referred for metastatic MM or UM in the same centers before the approval of anti-CTLA-4 and anti-PD-1 mAbs and were treated with various chemotherapy regimens as first-line treatment.

## 2. Patients and Methods

### 2.1. Study Design

A multicenter retrospective study was performed in the dermatology departments of 25 general- and university- hospitals in France. Patients with melanoma were identified using the French *«*Association for Developing Informatics in Cytology and Anatomic Pathology*»* (ADICAP) classification. Patients with UM and MM were secondarily selected.

Inclusion criteria were the following: (i) stage III-C to IV (advanced) mucosal or uveal melanoma, whose diagnosis was histologically confirmed either on the primary tumor or on a metastasis; (ii) patients who received at least one infusion of anti-CTLA-4 or anti-PD-1 mAbs used either as first or second line, between 2008 and 2016; (iii) patients in the chemotherapy subgroup were treated with at least one cycle of chemotherapy, including carboplatin, fotemustine, dacarbazine, or temozolomide between 2000 and 2016 without further immunotherapy or BRAF or MEK inhibitors; (iv) minimal follow-up of 3 months after the first cycle of treatment in alive patients; and (v) radiologic assessment of tumor response at Week 12 with CT scan. Additionally, patients with brain metastasis were evaluated using brain MRI.

Patients treated with a combination of anti-CTLA-4 and anti-PD-1 were excluded. This association was not tested in our study due to the low number of data collected on patients enrolled in a clinical trial.

Clinical, histological, and radiological data were retrieved from medical records. They included gender, age, melanoma subtype, stage, site(s) of metastatic disease at the initiation of systemic treatment, presence of BRAF, NRAS, or KIT mutations, treatment regimen, response to first-line chemotherapy or immunotherapy, and survival status at the last follow-up visit.

Anti-CTLA-4 mAb (ipilimumab) was administered at a dose of 3 mg/kg intravenously every 3 weeks for a total of four infusions. Anti-PD-1 mAb was given at a dose of 3mg/kg every two weeks for nivolumab, and at a dose of 2mg/kg every three weeks for pembrolizumab.

### 2.2. Outcomes and Assessments

The primary endpoint was the objective response rate at Week 12. Tumor response was assessed according to the Response Evaluation Criteria in Solid Tumor (RECIST) guidelines version 1.1 [23]. Objective response rate was defined as the proportion of patients who achieved complete or partial response at Week 12. Patients who received one or more doses of therapy without subsequent radiographic evaluation were excluded.

Planned secondary endpoints were: (i) disease control rate, as the proportion of patients who achieved at least stabilization at Week 12; (ii) response rate to anti-CTLA-4 mAb versus anti-PD-1 mAbs; (iii) response rate to immunotherapy (Anti-CTLA-4 mAb and anti-PD-1 mAbs) depending on whether it was used as first-line treatment or after an initial chemotherapy regimen; (iv) progression-free survival (PFS); (v) overall survival (OS); and (vi) rate of serious adverse events. Toxicity was graded according to the Common Terminology Criteria for Adverse Events (CTCAE) version 4.0 (NCI 2009) [24]. Since more than 50% progression was observed at Month 3, the median progression-free survival was less than 3 months in all groups. The exact progression time was unknown within the first months; consequently comparison of PFS between groups was not possible and this outcome is not shown.

LDH was not recorded in the present study, since this investigation was not systematically performed by investigators.

The study was approved by the Institutional Review Board of Rouen University Hospital (CCTIRS N 16766).

### 2.3. Statistical Analysis

Due to different characteristics, MM and UM subgroups were analyzed separately.

Baseline characteristics are presented as frequencies and percentages for categorical variables and as median, and mean ± standard deviation or median (interquartile range (IQR)) for quantitative variables.

The main analysis was the comparison of 3-month response rates between the two treatment groups (immunotherapy versus chemotherapy) and between anti-CTLA-4 and anti-PD-1 using the central Fisher's exact test in patients with MM and in those with UM.

Overall survival was defined as the time from initiation of the first immunotherapy or chemotherapy to time of the last follow-up visit or death. Progression-free survival was calculated from the time of initiation of immunotherapy or chemotherapy to the time of documented disease progression, last follow-up visit (in responders or in patients with stable disease), or death. Survival distributions were estimated using the Kaplan Meier method. Comparisons between treatment groups were made using Cox models.

The secondary analysis was a Cox model adjusted for main prognostic factors (lung, liver, nodular and cutaneous metastasis at initiation of treatment, age, and rank-transformed delay from diagnosis of primary melanoma to initiation of first-line treatment for UM). Multiple imputations by chained equations, provided by the “mice” package of the R statistical software, were used to impute delay from diagnosis of primary melanoma to initiation of first-line treatment for multivariate models of UM.

All statistical tests used the two-sided 0.05 level as their significance threshold.

Analyses were performed on R statistical software (release 3.4.3, the R Foundation for Statistical Computing, Vienna, Austria).

## 3. Results

### 3.1. Demographic and Clinical Characteristics

Between January 2000 and December 2016, 439 patients from 25 centers in France were retrospectively included in the study, 229 (52%) with MM and 210 (48%) with UM. Patients' characteristics are detailed in Table 1. The mean ± SD age of patients with MM at the time of chemotherapy or immunotherapy initiation was 68.1 ± 11.9 years and that of patients with UM was 64.9 ± 12.2 years. Main locations of primary MM were head and neck (n=89), anorectal (n=64), vulvovaginal (n=59), conjunctiva (n=9), and urologic (n=8). The UM subgroup included 203 patients with choroid melanoma and 7 with other localizations (excluding conjunctiva). Median delay from diagnosis of primary melanoma to initiation of first-line treatment ranged from 16.5 to 59.0 months depending on type of melanoma and treatment subgroups (Table 1). The median follow-up duration of the whole population was 8.48 months [IQR=5.09-14.0] and that of alive patients was 10.2 months [IQR=6.0-18.1]. Main metastatic locations were liver (n=258), lung (n=180), lymph nodes (n=161), skin (n=135), bone (n=71), brain (n=37), and other sites (n=73). Comparison of pretreatment characteristics between patient subgroups showed that patients with MM treated with immunotherapy had a significantly lower frequency of liver metastasis (p=0.016) than patients treated by chemotherapy (Table 1). In the subgroup of patients with UM, lung metastasis was significantly more frequently observed in patients treated with immunotherapy than in those treated with chemotherapy (p=0.001) (Table 1).

### 3.2. Treatments

**Figure 1:**
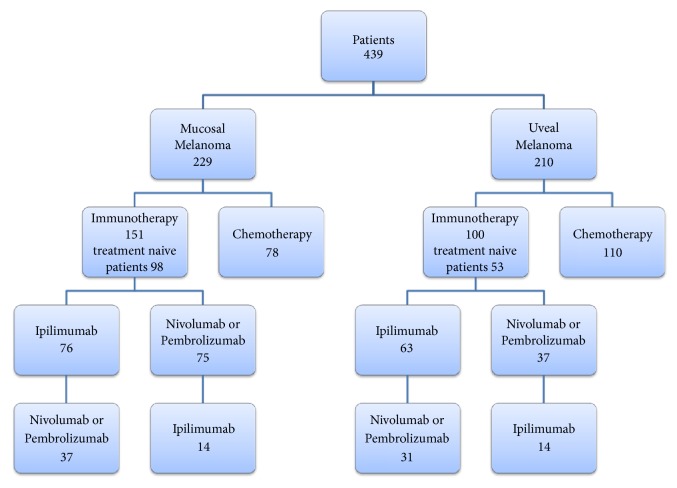
Flow chart of the study.

Seventy-eight patients with MM were initially treated with chemotherapy (34.1%) and 151 with immunotherapy (65.9%). In this latter group, 98 (64.9%) patients received immunotherapy as first line, and 53 (35.1%) patients were treated with subsequent immunotherapy after failure of a previous chemotherapy. Out of the 151 MM patients treated with immunotherapy, 76 (50.3%) received initial ipilimumab and 75 (49.7%) received initial nivolumab or pembrolizumab. Fourteen of these 75 patients were secondarily switched to ipilimumab for those who started with anti-PD-1 or vice versa (n=37) (Figure 1).

One-hundred and ten patients with UM were treated with initial chemotherapy (52.4%) and 100 with immunotherapy (47.6%). In this latter group, 52 patients had first-line immunotherapy, and 48 patients were treated with immunotherapy after the failure of previous chemotherapy. Sixty-three out of the 100 (63%) UM patients received initial ipilimumab, and 37 received initial nivolumab or pembrolizumab. Fourteen of these 37 patients were secondarily switched to receive ipilimumab or vice versa (n=31) (Figure 1).

### 3.3. Assessment of the Primary Endpoint: Objective Response Rate

#### 3.3.1. Mucosal Melanoma

At Week 12, an objective response was observed in 18 of 151 (11.9%, 95% CI: 7.2%-18.2%) MM patients treated with immunotherapy (including 6 patients with complete response and 12 with partial response) and in 11 of 78 (14.1%, 95% CI: 7.3%-23.8%) patients treated with chemotherapy (including 2 patients with complete response and 9 with partial response). The immunotherapy to chemotherapy odds ratio was estimated at 1.10 (95% CI: 0.59-2.06, p=0.87).

Because of the low response rate, the adjustment could not be performed on main prognostic factors.

#### 3.3.2. Uveal Melanoma

A tumor response was observed in 4 patients (3.6%; 95% CI: 1.0-9.0%) treated with chemotherapy (including 2 patients with complete response and 2 patients with partial response) and in no patient treated with immunotherapy (0%; 95% CI: 0.0-3.6%).

### 3.4. Assessment of Secondary Endpoints

#### 3.4.1. Disease Control Rate

*Mucosal Melanoma.* Taking into account the achievement of stable disease, which was observed in 27 patients (17.9%) treated with immunotherapy and 13 patients (16.7%) treated with chemotherapy, the disease control rate was 29.8% in patients treated with immunotherapy and 30.8% in those treated with chemotherapy.

*Uveal Melanoma*. Stable disease was observed in 29 patients (26.4%) treated with chemotherapy and in 32 (32%) patients treated with immunotherapy, corresponding to disease control rates of 30.0% (95% CI: 21.6-39.5%) in patients treated with chemotherapy and 32% (95% CI: 23.0-42.1%) in those treated with immunotherapy.

#### 3.4.2. Response Rate to Anti-CTLA-4 versus Anti-PD-1 Antibodies

*Mucosal Melanoma*. Fifteen of the 18 responses in patients treated by immunotherapy were observed with anti-PD1 mAbs, versus 3 with anti-CTLA-4, corresponding to objective responses rates of 20% (95% CI: 11.6%-30.8%) and 3.9% (95% CI: 0.8%-11.1%), respectively (p=0.01).

*Uveal Melanoma. *No response was observed in patients treated with immunotherapy. Stable disease was observed in 19 of the 63 patients (30.2%) treated with anti-CTLA-4 versus 13 of the 37 patients (35.1%) treated with anti-PD1 mAbs (p=0.76).

#### 3.4.3. Response Rate to Immunotherapy Depending on First or Further Lines of Treatment

*Mucosal Melanoma*. At Week 12, a tumor response was observed in 14 of 98 (14.3%; 95% CI: 8.70%-22.8%) treatment-naive MM patients (corresponding to 14 patients treated with anti-PD1), versus 4 of 53 (7.5%; 95% CI: 2.1%-18.2%) patients who were previously treated (p=0.34).

#### 3.4.4. Survival

**Figure 2:**
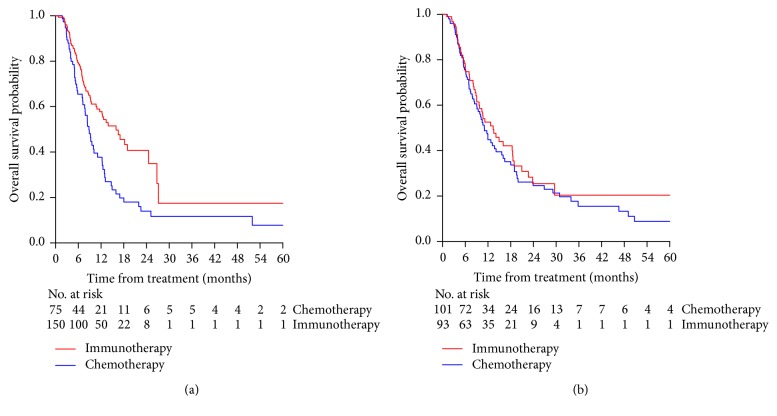
Kaplan Meier estimate of overall survival of patients with mucosal melanoma (panel (a)) or uveal melanoma (panel (b)) treated by immunotherapy (red curve) or chemotherapy ( blue curve).

*Mucosal Melanoma*. The analyses were performed on 225 patients due to missing data on follow-up dates. The unadjusted OS of patients treated with immunotherapy was significantly longer than that of patients treated with chemotherapy, HR= 0.56 (95% CI: 0.39-0.80), p= 0.001, with a median OS of 15.97 months [IQR=6.89-27.11] in patients treated by immunotherapy and 8.82 months [IQR=5.02-14.92] in those treated with chemotherapy. The one-year OS rates of patients treated with immunotherapy and chemotherapy were 57.8% (95% CI: 49.5-67.5%) and 37.8% (95% CI: 27.5-51.8%), respectively (Figure 2(a)).

After adjusting for the main prognostic factors (lung, liver, nodular and cutaneous metastases, and age), the OS of patients treated with immunotherapy was significantly longer than that of patients treated with chemotherapy, HR= 0.62 (95%CI: 0.43-0.91), p=0.014.

*Uveal Melanoma*. The analyses were performed on 194 patients due to 16 missing data on follow-up dates.

The unadjusted OS of patients treated with immunotherapy was not significantly different from those of patients treated with chemotherapy, HR=0.88 (95% CI: 0.61–1.26), p=0.48, with a median OS of 13.38 months [IQR=6.03-29.57] versus 11.02 months [IQR=6.13-23.93]. The one-year OS rates of patients treated with immunotherapy and chemotherapy were 52.5% (95% CI: 40.1 – 63.0%) versus 44.3% (95% CI: 34.2 – 54.8%), respectively (Figure 2(b)).

After adjusting for main prognostic factors (lung, liver, nodular and cutaneous metastases, age, and rank-transformed delay from diagnosis of primary melanoma to initiation of first-line treatment), the OS of patients with UM was not significantly different from that of patients treated with chemotherapy, HR= 0.98 (95% CI: 0.66 – 1.44) (p=0.92).

### 3.5. Safety

Forty-four grade 3 or 4 adverse events were reported in 34 of the 251 MM and UM patients treated with immunotherapy (26 severe adverse events in 20 patients treated with anti-CTLA-4, and 18 severe adverse events in 14 patients treated with anti-PD1). Additionally, one patient died from an ulcerative colitis.

## 4. Discussion

This study showed that the highest response rate was observed in patients with MM treated with anti-PD-1, with nearly 20% (95% CI: 11.6%-30.8%) of responses relative to 3.9% (95% CI: 0.8%-11.1%) with anti-CTLA-4 mAb and 14.1% (95% CI: 7.3%-23.8%) with chemotherapy. The patients treated with immunotherapy had longer adjusted and unadjusted OS than patients treated by chemotherapy (15.97 versus 8.82 months).

On the contrary, only extremely low response rates were observed in patients with UM in all groups. Accordingly, no significant difference in overall survival could be observed between these two treatment subgroups. Disease control was observed in 32% of UM patients, which was close to that observed in patients treated by chemotherapy (30.0%). These findings support the first-line use of anti-PD-1 mAbs in MM patients more than in patients with UM.

The response rates observed in the present study were close to those reported in previous series of MM patients treated with anti-CTLA-4 or anti-PD-1 mAbs, which ranged between 7% and 12% with anti-CTLA-4 [19, 20] and between 19% and 23% with anti-PD-1 mAbs [21, 25–27], but which were lower than response rates reported in cutaneous melanoma [13].

Recently, D'Angelo et al. reported a 37.1% response rate in 35 patients with MM treated with an association of anti-CTLA-4 and anti-PD-1, suggesting the interest of this combination [28]. This association was not tested in our study due to the low number of data collected on patients enrolled in a clinical trial.

Similar to previous published series, we observed an extremely low rate of response in patients with UM, regardless of treatment. Indeed, response rates between 0 and 4.5% have previously been reported with anti-CTLA-4 [29–31], 3.6 to 8% with anti-PD1 [22, 32], and 0% to 8% with chemotherapy [33–35]. The exact reasons for such differences in response to treatment between uveal and mucosal melanoma subtypes remain unclear.

The present study has several strengths. First, it included a large number of patients, whereas only limited series of 7 to 86 patients with MM or UM treated with immunotherapy have been reported to the best of our knowledge [18, 20, 28]. Second, the large multicenter characteristic of our population makes a representativity bias unlikely. All patients were treated homogeneously with the same regimen of immunotherapy, according to FDA and EMA approval of anti-CTLA-4 and anti-PD1 mAbs [36–38].

The main limitation of this study is its retrospective character and the absence of randomization, with an indication bias penalizing immunotherapy as it may be prescribed in second line. Currently, to the best of our knowledge, no randomized controlled trial has been performed in patients with MM or UM, which is related to the rarity of these melanoma subtypes. That is why we assessed tumor response to various chemotherapy regimens on a historical series of UM and MM patients treated before the approval of immunotherapy. Since the characteristics of the two subpopulations were not identical, we adjusted our results for main prognostic factors in secondary analyses. Despite the retrospective character of the study, few patients were lost to follow-up.

No centralized review of radiological response was performed. However, the use of RECIST criteria means that a differential information bias due to inter-rater discordance between radiologists is unlikely.

This study confirms the poor efficacy of anti-CTLA-4 mAb for MM treatment and the better efficacy of anti-PD-1. Immunotherapy may improve OS in the MM group with a possible residual indication bias. The efficacy of immunotherapy in patients with UM remains disappointing and close to that observed with conventional chemotherapy.

## Figures and Tables

**Table 1:** Baseline characteristics of study patients.

	Mucosal melanoma			Uveal melanoma		
	immunotherapy	chemotherapy		immunotherapy	chemotherapy	
Number of patients	151	78		100	110	
Gender						
Female, no(%)	106 (70.2)	58 (74.36)	*p=0.54*	57 (57.0)	58 (47.27)	*p=0.58*
Male, no(%)	45 (29.8)	20 (25.64)		43 (43.0)	52 (52.73)	
Mean age (y) at metastasis diagnosis (SD)	66.3 (13.0)	68.2 (12.3)	*p=0.39*	65.0 (12.5)	62.9 (12.4)	*p=0.22*
Melanoma localization, no(%)						
Uveal						
Choroid	-	-		96 (96)	107 (97.3)	*p=0.71*
Other	-	-		4 (4)	3 (3)	
Conjunctiva	6 (4)	3 (3.8)	*p=0.99*	-	-	
Oral cavity	59 (39.1)	30 (38.5)		-	-	
Digestive	42 (27.8)	22 (28.2)		-	-	
Urologic	6 (4)	2 (2.6)		-	-	
Gynecological	38 (25.2)	21 (26.9)		-	-	
Mutation, no/denom(%)^*∗*^						
BRAF	6/143 (4.2)	-		0/66	-	
CKIT	20/127 (15.7)	-		0/56	-	
NRAS	11/99 (11.1)	-		0/50	-	
First-line immunotherapy, no(%)						
Anti-CTLA4	76 (50.3)	-		63 (63.0)	-	
Anti-PD1	75 (49.7)	-		37 (37.0)	-	
First-line chemotherapy, no(%)						
Fotemustine	-	5 (6.4)		-	27 (24.5)	
Dacarbazine	-	44 (56.4)		-	56 (50.9)	
other	-	29 (37.2)		-	27 (24.5)	
Treatment before immunotherapy, no (%)	53 (35.1)	-		48 (48.0)	-	
Metastasis at the first treatment, no(%)						
Brain	14 (9.3)	7 (9)	*p=1.00*	9 (9.0)	7 (6.4)	*p=0.65*
Liver	44 (29.1)	36 (46.2)	*p=0.016*	79 (79.0)	99 (90)	*p=0.043*
Cutaneous	80 (53)	32 (41)	*p=0.11*	15 (15.0)	8 (7.3)	*p=0.12*
Lung	67 (44.4)	37 (47.4)	*p=0.76*	48 (48.0)	28 (25.5)	*p=0.001*
Bones	22 (14.6)	12 (15.4)	*p=1.00*	22 (22.0)	15 (13.6)	*p=0.16*
Lymph node	91 (60.3)	41 (52.6)	*p=0.33*	18 (18.0)	11 (10)	*p=0.14*
Other	18 (11.9)	22 (28.2)	*p=0.003*	7 (7.0)	26 (23.6)	*p=0.003*
Delay between initial diagnosis and first treatment, month median (Q1; Q3)^*∗*^	21.4 (9.8;41.2)	16.5 (8.9; 37.2)	*p=0.30*	59.0 (25.2;120.3)	36.5 (21.1; 75.7)	*p=0.02*

^*∗*^ Many missing data.

## Data Availability

The clinical data used to support the findings of this study are restricted by the French National Commission on Information Technology and Freedom (French acronym: CNIL) in order to protect patient privacy. Data are available from Prof. Pascal Joly (Rouen University Hospital, Rouen, France) for researchers who meet the criteria for access to confidential data.
